# Implementation context for addressing social needs in a learning health system: a qualitative study

**DOI:** 10.1017/cts.2021.842

**Published:** 2021-08-31

**Authors:** Ryan P. Theis, Katherine Blackburn, Gloria Lipori, Christopher A. Harle, Michelle M. Alvarado, Peter J. Carek, Nadine Zemon, Angela Howard, Ramzi G. Salloum, Elizabeth A. Shenkman, the UF CTSI Learning Health System Program

**Affiliations:** 1 Department of Health Outcomes and Biomedical Informatics, University of Florida, Gainesville, FL, USA; 2 Learning Health System Program, Clinical and Translational Science Institute, University of Florida, Gainesville, FL, USA; 3 Department of Pharmaceutical Outcomes and Policy, University of Florida, Gainesville, FL, USA; 4 Department of Industrial and Systems Engineering, University of Florida, Gainesville, FL, USA; 5 Department of Epidemiology, University of Florida, Gainesville, FL, USA.

**Keywords:** social determinants of health, electronic health records, implementation science, qualitative research

## Abstract

**Introduction.:**

Unmet social needs contribute to growing health disparities and rising health care costs. Strategies to collect and integrate information on social needs into patients’ electronic health records (EHRs) show promise for connecting patients with community resources. However, gaps remain in understanding the contextual factors that impact implementing these interventions in clinical settings.

**Methods.:**

We conducted qualitative interviews with patients and focus groups with providers (January−September 2020) in two primary care clinics to inform the implementation of a module that collects and integrates patient-reported social needs information into the EHR. Questions addressed constructs within the Theoretical Framework for Acceptability and the Consolidated Framework for Implementation Research. Data were coded deductively using team-based framework analysis, followed by inductive coding and matrix analyses.

**Results.:**

Forty patients participated in interviews, with 20 recruited at the clinics and 20 from home. Two focus groups were conducted with a total of 12 providers. Factors salient to acceptability and feasibility included patients’ discomfort answering sensitive questions, concerns about privacy, difficulty reading/understanding module content, and technological literacy. Rapport with providers was a facilitator for patients to discuss social needs. Providers stressed that limited time with patients would be a barrier, and expressed concerns about the lack of available community resources.

**Conclusion.:**

Findings highlight the need for flexible approaches to assessing and discussing social needs with patients. Feasibility of the intervention is contingent upon support from the health system to facilitate social needs assessment and discussion. Further study of availability of community resources is needed to ensure intervention effectiveness.

## Introduction

Social determinants of health (SDoH) – defined as conditions in the physical and social environments “in which people grow, live, work, and age” – contribute to growing disparities in disease burden and rising health care costs [1–4]. Factors such as economic and housing instability, educational disparities, inadequate nutrition, and unreliable transportation can significantly influence population health, and the social needs of individual patients represent important points of intervention for policymakers and health-care providers [5].

In the USA, health system initiatives by the Centers for Medicare and Medicaid Services, state Medicaid programs, and Kaiser Permanente have used a range of patient- and population-level approaches to address social needs, including the tailoring of care plans and enhanced community partnerships [6–9]. For example, Medicaid accountable care organizations in Massachusetts work with community partner organizations to coordinate care for individuals who require behavioral health care or long-term services and supports [8].

Studies report evidence that interventions to collect, integrate, and use the information on patients’ social needs may be feasible in clinical settings [10–17] and effective in connecting patients with community resources [17–19], reducing social needs [18,19], and improving health outcomes [20]. In a systematic review, Gottlieb et al. [21] reported that such interventions could improve the identification of social needs and referrals to social agencies. However, evidence for their effectiveness in increasing patient uptake of social referrals and improving health outcomes is mixed. Improvements in health behaviors, physical and behavioral health, and quality of life found in some studies are likely specific to the context of program implementation.

Furthermore, despite evidence that policy interventions targeting social needs are effective, there remain challenges to implementing them in clinical and community settings [22–24]. The success of these programs requires a reliable system for collecting and integrating information on patients’ social needs. In 2013, an Institute of Medicine committee recommended the routine collection of a comprehensive set of patient-reported measures, which can be incorporated into electronic health record (EHR) systems for use in healthcare encounters [25,26]. The measures use questions from validated instruments to address domains relevant to behavioral health and social needs, which are incorporated into the Protocol for Responding to and Assessing Patient Assets, Risks, and Experiences instrument, which in turn forms the basis of templates used in several EHR systems [13,27].

Among these, the “Healthy Planet” module developed for Epic© by the Oregon Community Health Information Network is being implemented and tested in 30 community health centers nationally [28–30]. As part of a widely used EHR system, the Epic module can be leveraged to introduce patient-level social needs information into learning health systems (LHSs), which are health care delivery systems that combine research, data science, and quality improvement using clinical information collected at the point of care [31]. Yet, there remain gaps in knowledge about how to best integrate the module into clinical settings, taking into account the perspectives of clinicians and patients.

This article addresses these questions within the context of the University of Florida (UF) Health system, which coordinates the OneFlorida Clinical Research Consortium – one of the nation’s 13 PCORnet® clinical data research networks [32]. We present qualitative findings from a translational pilot project conducted by the UF Clinical and Translational Science Institute (CTSI) and its LHS program to inform program planning and implementation of the module in two UF Health clinics. Using workflow studies, interviews with patients, and focus groups with clinicians and staff at the study clinics, we elicited perceptions on acceptability and feasibility of the module and modes for administering it, and sought to understand anticipated barriers and facilitators to implementation.

## Materials and Methods

### Study Setting and Intervention

Workflow studies, patient interviews, and provider focus groups were conducted in two family medicine clinics in the UF Health system, located in North-Central Florida. Both clinics serve racially diverse patient populations, with 41% African-American, 30% covered by Medicaid, and 14% uninsured. Hispanic patients represent 4% of each clinic’s population.

For the purpose of this study, the *intervention* represents the combined process of social needs assessment, patient–clinician discussion, and referral to services for adult patients of the study clinics. The module includes questions across several domains, including physical activity, stress and anxiety, social support and activities, religious affiliation, marital status, intimate partner violence (IPV), difficulty affording basic needs, food insecurity, educational attainment, transportation difficulties, alcohol consumption, substance use, and housing challenges. Question wording is written at the eighth grade reading level. Once the module is completed, it becomes part of the patient’s EHR, and providers can use the information to tailor discussion with the patient and make referrals to community resources. At the time of this study, the module had been implemented in both clinics in a limited capacity (accessible only to health coaches), but no formalized processes were in place for social needs discussion or referral.

For this study, we considered the module domains, wording of questions and response options, and provider-facing EHR visualization to be core components of the intervention that are not subject to change. Peripheral components that can be adapted to fit local settings include the mode of administration (i.e., how, where, when, and with whom the patient responds to module questions) and follow-up practices (i.e., discussing social needs with patients and making community referrals).

We programed a REDCap version of the module for patients to complete during interviews using an electronic tablet.[33] This allowed patient responses to be available for research purposes, but not as part of their medical record. For provider focus groups, we produced screenshots of module questions as they appeared in the REDCap version and in the provider-facing EHR interface.

### Study Approach

Study design was guided by two conceptual frameworks: (1) the Theoretical Framework of Acceptability (TFA) [34], reflecting the extent to which patients consider the intervention to be appropriate, based on experienced or anticipated cognitive and emotional responses to the intervention; and (2) the Consolidated Framework for Implementation Research (CFIR), which can help identify factors that influence the implementation of multilevel interventions from the perspective of providers [35]. We addressed factors in four CFIR domains: (1) intervention characteristics; (2) outer setting; (3) inner setting; and (4) characteristics of individuals. We did not address the CFIR process domain, as processes for implementation were not yet developed. The TFA and CFIR models informed study design and interpretation of findings for the patient and provider participants, respectively.

To address implementation context specific to the study clinics, we first conducted workflow studies to identify potential points of administering the intervention. Workflow studies use flowcharts to understand the sequential and parallel steps in a process [36]. A team of industrial and systems engineers visited the clinics to map their workflows, interview clinicians about the patient care process, and conduct time studies. The resulting workflow maps were used in provider focus groups to facilitate discussion tailored to each clinic.

We also engaged with OneFlorida/UF Citizen Scientists to offer feedback and assistance with study design and data collection. Citizen Scientists are community members who contribute to the research process by engaging with researchers to improve the quality of health care [37]. The Citizen Scientists hold regular, paid positions within the UF CTSI and are members of the LHS Program Operations Committee. For this study, two Citizen Scientists reviewed study tools during development and participated as cointerviewers for patient interviews (NZ, AH).

### Participants

We recruited a convenience sample of 20 patients (10 from each clinic) to participate in interviews conducted in the clinic (*clinic interview group*), and an additional 20 patients (10 from each clinic) who had a history of being hospitalized at least twice in the last year, whom we approached using home contact information (*home interview group*). Clinic interviews were conducted at the study sites in person. On days that interviewers were present on-site, clinic staff described the study to patients after their scheduled visit. Patients who expressed interest were then taken to a private room and introduced to the interviewers, who explained the study and obtained informed consent.

The home interview group was part of the original protocol (developed prior to COVID-19), and allowed us to include patients with more complex health and social needs, who often face deficiencies in access to routine health care [38–40]. Health coaches and medical directors at each clinic generated a list of eligible patients, who were then mailed a letter from their clinic explaining the study. Patients identified for home interviews who did not opt out were contacted by clinic staff, and those who indicated interest were called by a study interviewer (KB) to set up an interview appointment.

For provider focus groups, recruitment within the study clinics was purposefully broad to include different clinical roles (e.g., physicians, nurses, social workers, clinic managers). All clinicians and staff at each clinic were contacted by a study coordinator by email to introduce the study and invite them to participate.

All study participants received $25 for their time.

### Data Collection

At the start of each patient interview, we presented the module questions to patients – either by asking them to complete the module on the tablet, or by verbally asking them the questions in cases where participants scored low for health literacy or were participating by telephone. We then posed questions to patients about their perspectives on the module using a semi-structured interview guide, which was reviewed by Citizen Scientists and revised to address their comments before fielding. The interview guide followed the TFA constructs of *affective attitude* (individual feelings about the intervention), *burden* (the effort required to complete the module), *perceived effectiveness* (perception that the module could effectively collect social needs information and that community referrals could improve health), and *self-efficacy* (the individual’s confidence in being able to respond to module questions and discuss social needs with providers). Patients were also asked for their perspectives on alternate modes for completing the module, including in-person assessment with providers in the clinic, using an electronic tablet in the waiting room, or using the UF Health online patient portal before their visit. All patient interviews were conducted in English, and no participants declined or were excluded due to language barriers.

Clinic interviews were conducted in person by study authors trained in qualitative interviewing (RT, KB, NZ, AH) in January and February 2020 at the clinic immediately following the patient’s scheduled visit. Each interview was conducted by two interviewers. Interviewers recorded self-reported information on the participant’s sex and race/ethnicity and administered the Rapid Assessment of Adult Literacy in Medicine – Short Form (REALM-SF) to assess health literacy [41]. The REALM-SF was included to describe the study population and as a tool to improve data collection. Interviewers offered assistance in completing the tablet version of the module to all patients with a REALM-SF score of seventh grade or lower.

Home interviews were conducted remotely by telephone or videoconference from July to September 2020 by a study author trained in qualitative interviewing (KB). Information was collected on the participant’s age, sex, and race/ethnicity by self-report. The REALM-SF was administered only to patients who participated via videoconference. Completion of the module was considered assisted in all remote interviews, with the interviewer reading the module questions and response options to the participant.

Videoconference focus groups were conducted in July 2020 for each of the two study clinics by a study author trained in focus group moderation (RT). Focus groups began by eliciting participants’ beliefs about SDoH and their awareness of the Epic module, followed by a slide presentation of the patient-facing and provider-facing versions of the module. We then explored provider perspectives on the intervention, focusing on constructs in four CFIR domains – *intervention characteristics* (evidence strength and quality, adaptability, and complexity); *outer setting* (external policies and incentives); *inner setting* (implementation climate); and *individual characteristics* (knowledge and beliefs about the intervention). To assess how the module could be integrated into the clinic’s setting, participants were shown the flowchart for their clinic developed during the workflow study.

Interviews and focus groups were audio-recorded and transcribed for review and analysis, and were conducted concurrently with data collection to permit iterative modifications to the interview guides and assess thematic saturation [42].

### Data Analysis

Interview and focus group transcripts were reviewed by a team of qualitative coders led by the investigators who collected the data (RT, KB). We followed a directed approach to content analysis and used a framework method for reviewing transcripts, which allows qualitative findings to be shared more readily with diverse stakeholders and facilitates more rapid development of implementation strategies [43–45]. Coders were trained in team meetings led by the first author (RT), who is an expert in qualitative research.

Structured templates were developed for review and summary of transcripts. The templates included domains derived from CFIR and TFA, emphasizing the acceptability of the intervention and implementation context. Each transcript was reviewed and summarized by an initial primary reviewer, and the resulting summary was then reviewed by a secondary reviewer against the original transcript. Secondary reviewers provided input on content missing from or extraneous to the summary, and for moving content to different domain fields. Notated summaries were exchanged between the primary and secondary reviewers until both indicated full agreement, with a third team member available to resolve discrepancies.

Summarized content was organized into participant-by-domain matrices for patient interviews and focus group-by-domain matrices for provider focus groups [46]. This approach permitted separate analyses of findings for each study clinic and across participant characteristics. For patient interviews, matrices explored the acceptability of the three components of the intervention (module content, assessment, and discussion). The first author (RT) then conducted inductive coding of summarized content to capture emerging themes most relevant for implementation. All inductive codes and definitions were reviewed against the content by the second author (KB) to ensure consistency in their application. Changes made to codes and code definitions during this iterative process were documented to establish an audit trail. Finally, we generated an aggregate matrix to triangulate findings on patient and provider preferences for intervention delivery.

## Results

Study findings are presented in three sections. Findings from patient interviews include information on patient characteristics and a thematic analysis of acceptability of the intervention, including the valence of acceptability and themes organized according to TFA constructs. Findings from provider focus groups include information on provider characteristics and a thematic analysis of acceptability and feasibility organized according to CFIR domains. Lastly, we present considerations for implementation specific to the study sites, which converge findings from patients and providers.

### Patient Interviews

A total of 40 patients participated in interviews (20 from each clinic), although one clinic interview was not audio-recorded due to equipment malfunction and was not included in the analysis. The number of interviews conducted was sufficient to achieve thematic saturation for the clinic and home interview groups separately. Table 1 shows the distribution of patient participants by selected characteristics. The majority of interview participants were female (74%), African-American (59%), and completed the module with assistance from interviewers (79%). Participant age was not routinely collected for patients in the clinic interview group; patients in the home interview group ranged in age from 26 to 71 years old (mean: 53 years).

**Table 1.** Patient interviews − participant characteristics

	Clinic interviews (N = 19)		Home interviews (N = 20)		Overall (N = 39)	
	**n**	**%**	**n**	**%**	**n**	**%**
Participant sex						
Female	14	74%	15	75%	29	74%
Male	5	26%	5	25%	10	26%
Participant race**/**ethnicity						
African-American, NH	11	58%	12	60%	23	59%
Latina	1	5%	0	0%	1	3%
White, NH	5	26%	8	40%	13	33%
Not reported	2	11%	0	0%	2	5%
Participant age^a^						
Less than 40 years old	–	–	3	15%	3	15%
40−59 years old	–	–	11	55%	11	55%
60 years or older	–	–	6	30%	6	30%
REALM-SF score category^b^						
High school	10	56%	3	100%	13	62%
Seventh to eighth grade	0	0%	0	0%	0	0%
Fourth to sixth grade	5	28%	0	0%	5	24%
Less than fourth grade	3	17%	0	0%	3	14%
Module administration						
Assisted	11	58%	20	100%	31	79%
Unassisted	8	42%	0	0%	8	21%

a Participant age was collected only from patients in the home interview group (n = 20).

b The REALM-SF was administered only to patients who participated in in-person or videoconference interviews (n = 21). One patient in the clinic interview group was not administered the REALM-SF.

Abbreviations: NH, non-Hispanic; REALM-SF, Rapid Assessment of Adult Literacy in Medicine – Short Form.

Among 21 patients who were administered the REALM-SF, 62% scored for health literacy at high school level or higher and 24% scored at fourth to sixth grade level. The remaining 14% scored at less than fourth grade level. Differences in health literacy between the clinic and home interview groups are not meaningful, as only three home interview participants completed the REALM-SF.

Direct quotations from patient interviews include information on the patient’s interview group (Clinic or Home), sex (F = female, M = male), and race/ethnicity (AA = African-American, W = White, NR = not reported). A respondent identification number specific to the patient interviews (ID) is also included to differentiate quotations between patients with the same characteristics.

#### Thematic analysis of patient acceptability

Coders first assigned a valence label to patient responses related to acceptability of the intervention, overall, and separately for the module questions, assessment, and discussion (Table 2). Valence represents the general level of acceptability the patient expressed about the intervention. Acceptability was highest for social needs discussion, with 74% of participants having positive views.

**Table 2.** Patient interviews − acceptability valence of social needs module content and follow-up practices

Acceptability valence	Intervention component				Quotations
	Questions	Assessment	Discussion^a^	Overall	
Positive	54%	59%	74%	62%	“You just never know what a person is going through. They are really good questions.” [Clinic, F, AA; ID #6] “I feel comfortable, because that’s the point of you coming here – to discuss these things.” [Clinic, F, AA; ID #5]
Neutral/both	33%	41%	15%	33%	“Some of these questions are pretty simple. I mean, they’re average questions.” [Home, M, W; ID #40] “As long as she don't try to make a mountain out of a molehill, I’m good… She should not try to… just say stuff. It makes me uncomfortable.” [Home, F, AA; ID #32]
Negative	13%	0%	8%	5%	“That question about your mortgage or rent or whatever, I wouldn't [answer]. And the food… I wouldn't want to share that.” [Home, F, AA; ID #33]

a One home interview participant did not respond to questions on acceptability of social needs discussion.

Abbreviations: AA, African-American; F, female; ID, respondent identification number; M, male; W, White.

Table 3 shows the most salient acceptability themes that emerged in patient interviews, organized according to their relevance to TFA constructs of affective attitude, burden, perceived effectiveness, and self-efficacy.

**Table 3.** Patient interviews − acceptability of the social needs intervention, themes by TFA construct

TFA Construct	Theme definition	Example quotation
Affective attitude		
Rapport with provider (n = 21)	Acceptability of social needs interventions depends on the interaction and relationship that patients have with their provider.	“It just depends on who your doctor is. You got to have a relationship or a rapport with your doctor. And if you're not able to trust your doctor, it doesn't matter, if you have to do the questionnaire or not. People are not going to be truthful.” [Clinic, F, AA; ID #3]
Life changes (n = 13)	Assessing social needs more frequently is important because a patient’s life condition can change.	“A lot can change in six months… You do not have any updates if you just do it once a year. You're still relying on what the questionnaire said at the beginning of the year. Your situation might have changed in a couple of months.” [Home, F, AA; ID #21]
Privacy (n = 9)	Acceptability of social needs interventions depends on the privacy and confidentiality of answers to social needs questions or discussions about social needs.	“That’s something that once it’s on [the portal]… someone can see it. This is between me and my doctor. You do it on the Internet, there’s a chance that that can get out there to somebody else. You got billing department. You got every doctor I see on that portal. [Clinic, M, W; ID #7]
Burden		
Discomfort (n = 12)	Patients may be less likely to disclose social needs because the questions or assessment cause discomfort (are personal, sensitive, or trigger emotions).	“My daughter’s 20, and we still struggle… I know if you ask my daughter, she would decline because she wouldn't want to be honest and truthful when it would hurt. Hurts when you answer the questions sometimes… You're like oh, wait, that makes me feel like failing as a parent and stuff.” [Clinic, F, NR; ID #8]
Time limitations (n = 9)	Having meaningful social needs assessment or discussion is difficult if the process takes too much time, or if done in a context where the patient or provider feels rushed for time.	“There’s a lot of times depending on when you arrive at your appointment… I do not have time to answer questionnaires. I do not want the stress of trying to answer this and bring it back here… so I can give it to her on time. Those little things.” [Clinic, M, AA; ID #9]
Stigma (n = 8)	Patients may be less likely to disclose social needs due to concerns about being judged by providers or other patients.	“I’m at a point where I may need more [medication] but I am afraid to ask, so I don't ask for more… I do not want them to judge me…” [Home, F, AA; ID #21]
Perceived effectiveness		
Positive outcomes (n = 16)	Assessing and addressing social needs will lead to positive outcomes, such as "help", referrals, or positive health outcomes.	“If you hadn't had food, maybe they could say, okay, she could be suffering from you know being hungry… If you're behind on your mortgage and bills or something - could be related to stress, high blood pressure, stuff like that.” [Home, F, AA; ID #33]
Important for routine care (n = 15)	Assessing and/or acting on social needs is important for a patient’s normal or routine health care.	“Well, [the questions] were more about living, and that was important… I do not know why a doctor wouldn't be concerned about those questions.” [Clinic, F, W; ID #11]
Trust building (n = 13)	Assessing social needs builds trust or understanding between patient and provider, and may improve their relationship.	“The physicians will know exactly what you're going through, exactly what you're feeling, and then… they can be able to relate to you.” [Clinic, F, AA; ID #20]
Encouraging disclosure (n = 11)	Answering social needs questions or discussing social needs will encourage patients to disclose personal information they might otherwise withhold.	“Because [the provider] can get more out of it if he’s talking… person-to-person. And [patients will] tell you more how they feel about it, if questions was asked to them. [Home, F, AA; ID #29]
Self-efficacy		
Honesty (n = 14)	Patients will answer social needs questions meaningfully if they have a personal commitment to being honest or truthful.	“I'm not afraid to tell anything to my doctor because I'm an open book. And I'm [on] short time anyhow… So the more information they have the better can help me, you know.” [Home, M, W; ID #27]
Technology literacy (n = 13)	Patients may not use technical applications for social needs assessment because they don't understand or can't use the technology (e.g., computers, tablets).	“No, [I would not be comfortable with the technology] because I don't know about anything about a computer or a phone. Only thing I know is how to say ‘Hello’ and ‘Goodbye’.” [Home, F, AA; ID #21]
Difficult to read/understand (n = 10)	Patients may have difficulty reading or understanding social needs questions and/or understanding social needs discussion with providers.	“[Completing questions in the waiting room] would suck… My eyes are really bad and it’s hard for me to see…. I do not like to strain my eyes.” [Home, M, W; ID #40]

Abbreviations: AA, African-American; F, female; ID, respondent identification number; M, male; NR, not reported; TFA, Theoretical Framework of Acceptability; W, White.

##### Affective attitude

Patients felt that acceptability of the intervention would be most impacted by their relationship with providers, life changes that can impact the need for assistance, and concerns about the privacy and confidentiality of social needs information.

For 21 patients, acceptability was contingent on having rapport with a specific provider. Without this relationship, patients may be less likely to respond truthfully to the questions, volunteer information, or ask for assistance with problems. One participant described her provider as being “like a family member,” stating: “I can tell her anything I want to tell her, so I can talk to her like I want to talk to her. But I wouldn't do it with nobody else.” [Home, F, AA; ID #32]

Patients noted that social needs should be assessed regularly because changes often occur in a person’s physical or social conditions. One patient remarked that worsening of one’s life situation could be the *reason* for their visit to the doctor, as stress can affect clinical outcomes such as blood pressure:

“If one [person] can make you feel so bad, it will run your blood pressure up. And if [the doctor] keeps talking to you and asking… questions… he'll figure out that… they're running your blood pressure sky-high." [Home, F, AA; ID #29]

Concerns about privacy could discourage patients from disclosing information. These include concerns that module answers would be visible to other people, discussions could be overheard, doctors would be unable to keep certain answers private (e.g., through mandatory reporting), and spouses or partners may discover that the patient had disclosed instances of IPV. Patients’ perspectives on privacy varied with regard to different locations and delivery modes. For example, some felt that the patient portal negated concerns about being overheard in the clinic, while others believed that the portal would compromise confidentiality by giving other people access to their data. Likewise, some patients preferred to respond to the questions in their home because it allowed for greater privacy, while others reported having no privacy at home due to the presence of other family members.

##### Burden

Patients described the burden of the intervention in terms of discomfort with sensitive questions, limited time to complete the assessment or discuss issues with their doctor, and concerns of being judged by providers or other patients.

Twelve patients noted that questions that were “too personal” could discourage them from disclosing information. A patient’s own history of having (or not having) social needs had implications on both the patient’s level of comfort and the effort involved in answering the questions. For patients who indicated that the questions “did not apply” to them, completing the module was a fast and simple activity. The module was more challenging for patients who had a history of social needs, as it required responding to more follow-up questions and could trigger unpleasant memories. One patient stated that “it hurts when you answer questions,” adding: “I do not want to go back and relive everything all over again when they ask about personal stuff in the past.” [Clinic, F, NR; ID #8]

Four patients expressed hesitation about completing the module in the waiting room, as they would feel “rushed for time” to complete it. These patients preferred to complete the module at home to allow them “more time to think” about their responses. One patient noted that time limitations could affect the accuracy of responses, stating: “People would just fill out anything to get back to the back… It would be a rush thing… You'll get more accurate answers face-to-face." [Home, M, AA; ID #26]

Patients noted that stigma associated with social needs could also impact their willingness to disclose information. For example, some suggested that patients who consume alcohol may not admit that they drink, or that patients might be too embarrassed to disclose IPV. Others highlighted the relevance of perceived stigma in face-to-face encounters with providers; as one patient related, completing the module on the tablet was preferable to responding in person, as a patient would not have to “feel judged.” [Clinic, F, AA; ID #3]

##### Perceived effectiveness

Patients described the intervention as being important for routine health care. They perceived the assessment as an effective tool for prompting discussion on topics that patients might otherwise avoid. In addition to achieving positive social and health outcomes, discussion of social needs was seen as a way to build trust with providers.

Patients noted that completing the module by self-report (e.g., on the patient portal or a tablet) could encourage patients to disclose more information, help them remember to bring up issues with providers, and relieve some of their burden. As one patient stated, “Then you can get everything off your chest.” [Home, F, AA; ID #32]

Patients also noted that discussing social needs with providers can help providers to “know patients” better, take more time with them, and improve their communication. As one patient remarked, the intervention “makes for a more complete relationship” with providers. [Home, M, W; ID #27]

Patients identified housing, food, transportation, IPV, alcohol problems, and medications as the most impactful social needs to address. They indicated that, through improved access to community resources, the intervention could bring direct benefits to health. One patient related the benefits of social connections for alleviating stress: “Being involved… Having people care… You do need people just to hang out with just to relieve stress.” [Clinic, F, NR; ID #8]

##### Self-efficacy

Patients related that using the module would be easier for those who are committed to giving “honest” answers, are able and willing to use technological applications, and do not have difficulty reading or understanding module content.

Three patients described themselves as “an open book,” relating that they would answer module questions honestly in any context. Others had varied views of the context most conducive to honesty, with four relating that they would be more honest completing the module by self-report, and two stating that they would be more honest in face-to-face encounters.

Thirteen patients described themselves as “not comfortable” using technological applications, with some suggesting they would need assistance using the patient portal or tablet. Low technological literacy was the most frequently mentioned barrier to using the patient portal – an issue that was seen as more relevant for older patients.

Ten patients found the module questions difficult to answer or understand, which occurred due to difficulty reading and low health literacy. Four patients explained that they would need assistance from a provider to explain the questions to them, and for this reason expressed a preference for face-to-face or phone-based assessment.

### Provider Focus Groups

We conducted videoconference focus groups with clinicians and staff who worked at Clinic 1 (n = 7) and Clinic 2 (n = 5) (Table 4). Physicians and nurses were present at both focus groups. A social worker and clinical pharmacist were present at Clinic 1, and a clinic manager was present at Clinic 2. Most had been working in their role at the clinic for less than 5 years. The number of focus groups was dictated by the practical aims of this study, rather than by thematic saturation. Statements by specific providers include information on the provider’s clinic, role, and a respondent identification number specific to the provider focus groups (ID).

**Table 4.** Provider focus groups – participant characteristics

	Clinic 1		Clinic 2	
	**N**	**%**	**N**	**%**
Provider type				
Physician	3	43%	3	60%
Nurse	2	29%	1	20%
Social worker	1	14%	0	0%
Clinical pharmacist	1	14%	0	0%
Clinic manager	0	0%	1	20%
				
Years in role				
Less than 5 years	5	71%	3	60%
5−10 years	2	29%	0	0%
Greater than 10 years	0	0%	2	40%
				
Participant sex				
Female	6	86%	5	100%
Male	1	14%	0	0%
				
Participant race				
White	4	57%	3	60%
Black or African-American	2	29%	1	20%
Asian	1	14%	0	0%
Unknown	0	0%	1	20%

#### Provider acceptability and feasibility findings by CFIR domain

Table 5 lists the most salient themes related to acceptability and feasibility of the intervention that emerged in focus groups, organized according to CFIR domain and construct. With the exception of one nurse/health coach, providers were unaware of the Epic module prior to the study. Comments focused primarily on the constructs of evidence strength/quality and complexity, while the construct of adaptability was less salient, in part because the screener topics, question wording, and EHR interface were core components of the intervention that could not be adapted. With regard to outer setting, providers focused on patient needs and resources, which was not explicitly addressed in the moderator guide but emerged as a salient construct. While providers related that external support would be needed to implement the intervention, they offered few specifics on policies and incentives that could facilitate implementation. Comments about implementation climate in the inner setting focused on compatibility of the intervention with the clinic, addressing issues such as clinician capacity and limited time with patients. A significant portion of focus group content addressed the individual characteristics of providers, who offered comments on their own knowledge and beliefs about social needs interventions generally.

**Table 5.** Provider focus groups − acceptability/feasibility of the social needs intervention, themes by CFIR domain

CFIR domain	Theme definition	Example quotation
Intervention characteristics		
*Evidence strength/quality*		
SDoH and access	Effect that social determinants have on patients’ access to care	"When they don't have the basic needs of life, it’s hard to get into the clinic, it’s hard for us to take care of medical problems." [Clinic 1, Physician; ID #1
Positive outcomes	Positive effect that social needs intervention has on health and service outcomes	“[If patients] were able to get help towards [affordability of medications], and they start taking their medications regularly. Of course, you see an immediate improvement in the blood pressure." [Clinic 1, Nurse; ID #5]
*Complexity*		
Redundant with practice	Overlap of module social needs questions with current patient care practices	"I often ask some of the questions when they're relevant, but if I then had to go and like click over to the other section and fill it in… I do not know if it would be worth my while.” [Clinic 2, Physician; ID #8]
Workflow impact	Disruptions in clinical workflow resulting from social needs intervention	“[Patients] already have different issues that they are coming to see you with… Then you get behind and then everyone… gets upset of you because of your own clinic flow… I do not know, it just creates a lot for you." [Clinic 2, Physician; ID #8]
Outer setting		
*Patient needs/resources*		
Need for privacy	Patient needs for privacy that affect disclosure of social needs	"If we’re going to talk about child abuse or domestic violence, there’s like a reluctance to report out of fear… and DCF involvement." [Clinic 1, Social worker; ID #2]
Need for comfort	Patient needs for comfort that are impacted by social needs intervention questions or timing	"Some questions even just by asking the question, you can offend somebody… ‘Do you have trouble affording your medications?’ ‘What do you mean? Do I look like I have trouble affording my medications?'" [Clinic 1, Clinical pharmacist; ID #7]
Resource availability	Availability of community resources to address patients’ social needs	“There’s a lot of children programs out there for like schools and connecting them… And for elderly adults, I know there’s also programs for them too especially if [they have]… complex medical conditions.” [Clinic 2, Physician; ID #8]
Inner setting		
*Implementation climate (compatibility)*		
Clinician capacity	Capacity of clinicians to administer social needs questions	"Maybe the LPNs could sit down during a nurse visit and make time for those questions. I mean, we all can take turns, all of us can do it. It’s just a lot of patients. I don't see how one person can be responsible for those questions." [Clinic 1, Nurse; ID #5]
Time limitations	Limited time available to assess or discuss social needs with patients	“Time is the number one [barrier]… There’s a fear among physicians of… opening up a can of worms… So many of our patients have multiple of these issues that you would end up just kind of taking a lot of the whole visit." [Clinic 2, Physician; ID #11]
Individual characteristics		
*Individual knowledge/beliefs*		
Limited solutions	Provider belief that solutions for addressing social needs are limited	“[If you're] opening this up… there’s not really a lot you can do right then and [patients] also already have different issues that they are coming to see you with.” [Clinic 2, Physician; ID #8]
Encouraging disclosure	Provider belief that social needs intervention can prompt patients to discuss issues	"Just having it there and if they answer it, it’s probably the starting point for you to go into the conversation. And you’ll feel a little more comfortable going and talking about it if there’s already something there." [Clinic 1, Physician; ID #4]
Trust building	Provider belief that social needs intervention can build trust/rapport with patients	"When you talk to them one-on-one and you really develop a relationship, they do tend to tell you more information than they might tell the physician when the physician goes in." [Clinic 1, Nurse; ID #5]

Abbreviations: CFIR, Consolidated Framework for Implementation Research; DCF, Department of Children and Families; LPN, licensed practical nurse; SDoH, social determinants of health.

##### Intervention characteristics

Two issues emerged with regard to complexity of the intervention. First, providers found that some module questions were already populated in the EHR social history tab as part of regular care. One physician suggested that “cross-pollinating” information between the module and the EHR would help to reduce these redundancies [Clinic 2, Physician; ID #9]. Second, based on review of the workflow diagrams, providers noted that the intervention could cause delays and disruption in clinical workflow, as the effort required by the intervention could take up most of a patient’s visit or necessitate scheduling a separate visit.

##### Outer setting

Most comments relevant to the outer setting dealt with patient needs, resources, and characteristics. Providers indicated that patients may not disclose social needs in the presence of other family members, or if they are seen by a provider who is not their usual source of care. Patients with children may be reluctant to discuss food security, housing security, or domestic abuse for fear of being reported to the state Department of Children and Families. Providers expressed concerns about the low availability of community resources for their patients, including supportive housing and food resources. One physician remarked that, while resources for children and elderly patients are available, there is little in the community to support middle-age adult patients [Clinic 2, Physician; ID #8].

##### Inner setting

Providers raised concerns about the low capacity of clinicians to administer the module, noting that clinicians are “too busy” to help with assessment. They also noted that limited time with patients could result in low fidelity of implementation. As one physician noted, incorporating the module even as infrequently as once per year would be “very hard” due to the “extra responsibility” [Clinic 2, Physician; ID #8].

##### Individual characteristics

Providers believed that assessment could prompt patients to discuss social needs in one-on-one interactions with them, particularly when they have a good rapport. Furthermore, discussing social needs with patients was seen as a way to build trust. However, some providers believed that solutions for addressing social needs would be limited when more proximal concerns of the patient’s health take priority.

### Considerations for Implementation

To explore feasibility of implementing the intervention, we converged patient and provider perspectives, focusing on findings that point toward practical considerations and potential adaptations. Several themes were salient to both patients and providers, including the impact of time limitations in clinical encounters, the perceived positive outcomes of the intervention, the impact of privacy concerns on disclosure of social needs, the importance of rapport as both facilitator and outcome of the intervention, and the belief that the intervention itself could encourage disclosure.

Providers indicated the assessment should be done for every new patient visit, and then again annually. Patient preferences on frequency varied, with half recommending every 6 months or less often, and one-quarter recommending the assessment be completed at every visit. Both groups stated that reassessment should be contingent on the patient’s condition, and that patients can be asked at check-in if anything about their situation has changed since their last assessment.

Patients and providers remarked on the difficulty that some patients would have reading or understanding the module questions or using technology. Both concerns have a bearing on the mode and location for conducting the assessment, as they reduce the feasibility of completing the module on the patient portal or using a tablet in the waiting room. Providers acknowledged that some face-to-face assessment would be necessary, but added that having to "walk patients through it" would take up valuable encounter time. Proposed solutions included conducting the assessment as a separate visit a few days before the patient’s scheduled visit, having social workers assess patients in the hospital, and taking flexible approaches to workflow integration, such as beginning the assessment in the waiting room and ending in the exam room. These solutions would require external support for clinics to administer the module questions.

The question of which provider type was most appropriate for discussing social needs differed between patients and providers. While two-thirds of patients preferred having social needs discussion with a physician, providers at both clinics agreed that physicians were too busy for this role. Rather, social workers were considered the most appropriate for discussion, as they know best how to navigate the social support system and are more familiar with available community resources.

## Discussion

This study revealed key considerations for implementing an intervention to collect and use information on patients’ social needs in a LHS. Prior studies have found similar interventions to be feasible in the context of specific populations and settings [10–13,15–17]. There is evidence that collecting information on patients’ social needs using a tablet-based screening tool [17] and in the waiting room of a clinic [13] can be acceptable to patients, acknowledging the assistance that some patients may need to use electronic screening tools. Practices to collect and integrate social needs information into EHR can improve documentation of social history [10] and patients’ disclosure of sensitive information [12]. Holt et al. [16] reported that integrating patient contextual data into clinical care is supported by clinicians, while acknowledging the time constraints of adding a new process to clinical encounters.

Within the context of workflow in local clinics, providers stressed that limited time with patients would be a barrier to both in-person social needs assessment and discussion – a concern that has been reported in other health systems and settings [15,16,47]. Provider comments about the unavailability of certain types of community resources also align with reports in other contexts [15]. Findings point to the need for increased availability of community resources and a system for social referrals that are appropriate, accessible, and timely for patients. A more formalized system of enabling services, such as health education, language interpretation, and transportation, can result in increased utilization of routine and preventive care [48].

A key finding was that patient preferences for intervention delivery varied according to their needs and values. Findings point to a need to balance the benefits of regular assessment with the burden that patients may feel from frequent assessments that ask the same (and in many cases, sensitive) questions. Patients with cognitive or vision impairments can have difficulty reading or understanding the module questions, and are less likely to complete the module using a tablet, which aligns with findings reported in a study of a tablet-based social needs questionnaire [17]. Furthermore, for patients with low literacy, a provider-administered mode was not considered compatible with the workflow and resource limitations of the study clinics. Given that the stigma of low literacy can affect the quality of spoken interactions between patients and providers [49], our study’s findings on patient perceptions of stigma may have a bearing on the acceptability of provider-administered assessments. While many patients in our study expressed concerns about privacy, they did not have a shared preference for the context of social needs assessment or discussion. Some cited concerns that other people might see their responses on a tablet in the waiting room, while others stated they had no privacy at home and would not respond to the module using the patient portal.

The barriers to implementing social needs interventions may have differential influence on when, where, and with whom social needs assessment and discussion should take place. Our findings speak to the need for a flexible approach that accounts for the patient’s age, health and technological literacy, access to technology, relationship with providers, and prior history of social needs, while also minimizing disruptions to clinical workflow. For example, a clinic may promote the use of the portal to patients willing and able to use technology, allowing them to complete the module from home, and also build capacity to assist patients with low technological literacy or impairments. While this approach would be more complex to implement, the consequent improvement in reach and completeness of social needs information would more equitably promote improvements in diagnosis and treatment, shared decision-making, identification of behavioral risk factors, referrals to community resources, health system capacity to tailor services to population needs, and increased availability of information on patient context to researchers [26].

This study had several limitations. The study was conducted in two clinics within a single health system, focusing on adult patients. Findings, therefore, may not be fully transferable to other organizations or pediatric populations. For example, while Epic is used in all clinics and hospitals within UF Health, other OneFlorida sites use different EHR systems. Furthermore, as all patient interviews were conducted in English, the study was unable to assess the potential influence of language as a barrier to addressing social needs. While our decision to conduct interviews in English was appropriate to the study clinics (with only 4% of patients identified as Hispanic), findings may not be transferable to other clinics with a higher percentage of patients whose predominant language is not English.

Patient interviews were conducted in two stages, beginning with clinic interviews and followed by home interviews. The COVID-19 pandemic occurred between these stages, resulting in all clinic interviews being conducted in person, and all home interviews being conducted remotely. It is possible that the remote data collection mode, which lacks certain advantages of in-person interviewing (e.g., non-verbal cues, context for pauses), may have resulted in lower completeness and depth of content from home interviews. Furthermore, the inability to administer the REALM-SF in telephone interviews precluded a full exploration of the impact of health literacy on feasibility of the intervention.

Our convenience sampling approach for clinic interviews allowed us to improve participation rates and ensure we included the perspectives of patients who make outpatient visits. However, this may have affected representativeness of patients according to other factors. Additionally, as our methods for testing the module were limited to self-administration or verbal administration by study staff, we could not explore patient experiences with the full range of options for completing the module. While we elicited patient perspectives on completing the module with a provider or using the patient portal, we were limited in the extent to which conclusions could be made about these modes.

Furthermore, while efforts were made to ensure that provider focus groups represented all roles in each clinic, only one focus group included a clinic manager. Without representation of this important role, feasibility findings in relation to clinic workflow may have been less complete. The study scope also did not allow for engagement of health system leadership or community-based organizations, whose perspectives are important for understanding the implications of resource limitations. Lastly, as our analytic approach began with a directed content analysis and framework method, with subsequent inductive coding, this phased approach has limited the opportunity to uncover novel themes.

## Conclusion

This work represents the first step in UF CTSI efforts to establish the feasibility of using a social needs intervention in outpatient settings. The study revealed several factors, salient to both patients and providers, which highlight potential strategies to improve acceptability and feasibility. Among these, it is important to ensure that social needs information can be easily collected from patients with attention to considerations of comfort, privacy, and technology literacy, and discussed with patients in a format that minimizes disruption to clinical workflow. To account for differences in patient needs and values, the intervention should also be flexible with regard to when, where, and with whom social needs are elicited and discussed. Given limitations in local availability of community resources, additional study is needed to understand whether and the extent to which the social needs intervention can effectively provide needed social supports to patients and improve outcomes.

## Supplementary material

For supplementary material accompanying this paper visit https://doi.org/10.1017/cts.2021.842. click here to view supplementary material

## References

[1] Marmot M. Social determinants of health inequalities. Lancet 2005; 365(9464): 1099–1104. DOI 10.1016/S0140-6736(05)71146-6. 15781105

[2] World Health Organization. Closing the Gap in a Generation: Health Equity Through Action on the Social Determinants of Health. Final Report of the Commission on Social Determinants of Health. Geneva, Switzerland: World Health Organization; 2008.

[3] National Academy of Sciences, Engineering, and Medicine. Communities in Action: Pathways to Health Equity. Washington, DC: The National Academies Press, 2017. 28418632

[4] Artiga S , Hinton E. *Beyond Health Care: The Role of Social Determinants in Promoting Health and Health Equity* [Internet] 2018 [cited Jan. 27, 2021]. (https://www.kff.org/racial-equity-and-health-policy/issue-brief/beyond-health-care-the-role-of-social-determinants-in-promoting-health-and-health-equity/)

[5] Salerno J , Bogard K. What do social determinants of health determine? Journal of Urban Health 2019; 96(6): 794–794. DOI 10.1007/s11524-019-00402-z. PMC6904704 31792697

[6] Centers for Medicare and Medicaid Services (CMS). *Accountable Health Communities Model* [Internet] 2020 [cited Jan. 27, 2021]. (https://innovation.cms.gov/innovation-models/ahcm)

[7] Spencer A , Freda B , McGinnis T , et al. Measuring Social Determinants of Health Among Medicaid Beneficiaries: Early State Lessons. Trenton, NJ: Center for Health Care Strategies, Inc, 2016.

[8] Goff SL , Gurewich D , Alcusky M , Kachoria AG , Nicholson J , Himmelstein J. Barriers and facilitators to implementation of value-based care models in new Medicaid accountable care organizations in Massachusetts: a study protocol. Frontiers in Public Health 2021; 9: 645665. DOI 10.3389/fpubh.2021.645665. 33889558 PMC8055830

[9] Johnson SR. *In Depth: Hospitals Tackling Social Determinants are Setting the Course for the Industry* [Internet] 2018. [cited Jan. 27, 2021]. (https://www.modernhealthcare.com/article/20180825/NEWS/180809949)

[10] Beck AF , Klein MD , Kahn RS. Identifying social risk via clinical social history embedded in the electronic health record. Clinical Pediatrics 2012; 51(10): 972–977. DOI 10.1177/0009922812441663. 22511197

[11] Hassan A , Blood EA , Pikcilingis A , et al. Youths’ health-related social problems: concerns often overlooked during the medical visit. Journal of Adolescent Health 2013; 53(2): 265–271. DOI 10.1016/j.jadohealth.2013.02.024. 23643339

[12] Gottlieb L , Hessler D , Long D , Amaya A , Adler N. A randomized trial on screening for social determinants of health: the iScreen study. Pediatrics 2014; 134(6): e1611–e1618. DOI 10.1542/peds.2014-1439. 25367545

[13] Kusnoor SV , Koonce TY , Hurley ST , et al. Collection of social determinants of health in the community clinic setting: a cross-sectional study. BMC Public Health 2018; 18(1): 698. DOI 10.1186/s12889-018-5453-2. 29699539 PMC5921557

[14] Gage-Bouchard EA , Rawl SM. Standardizing measurement of social and behavioral dimensions of cancer prevention and control to enhance outreach and engagement in NCI-designated cancer centers. Cancer Epidemiology Biomarkers & Prevention 2019; 28(3): 431–434. DOI 10.1158/1055-9965.EPI-18-0794. PMC6401272 30670459

[15] Pinto AD , Bondy M , Rucchetto A , Ihnat J , Kaufman A. Screening for poverty and intervening in a primary care setting: an acceptability and feasibility study. Family Practice 2019; 36(5): 634–638. DOI 10.1093/fampra/cmy129. 30649280 PMC6781937

[16] Holt JM , Cusatis R , Asan O , et al. Incorporating patient-generated contextual data into care: clinician perspectives using the Consolidated Framework for Implementation Science. Healthcare 2020; 8(1): 100369. DOI 10.1016/j.hjdsi.2019.100369. 31445878

[17] Palakshappa D , Benefield AJ , Furgurson KF , et al. Feasibility of mobile technology to identify and address patients’ unmet social needs in a primary care clinic. Population Health Management 2021; 24(3): 385–392. DOI 10.1089/pop.2020.0059. 32924796 PMC8215428

[18] Garg A , Toy S , Tripodis Y , Silverstein M , Freeman E. Addressing social determinants of health at well child care visits: a cluster RCT. Pediatrics 2015; 135(2): e296–304. DOI 10.1542/peds.2014-2888. 25560448 PMC4306802

[19] Hassan A , Scherer EA , Pikcilingis A , et al. Improving social determinants of health: effectiveness of a web-based intervention. American Journal of Preventive Medicine 2015; 49(6): 822–831. DOI 10.1016/j.amepre.2015.04.023. 26215831

[20] Gottlieb LM , Hessler D , Long D , et al. Effects of social needs screening and in-person service navigation on child health: a randomized clinical trial. JAMA Pediatrics 2016; 170(11): e162521. DOI 10.1001/jamapediatrics.2016.2521. 27599265

[21] Gottlieb LM , Wing H , Adler NE. A systematic review of interventions on patients' social and economic needs. American Journal of Preventive Medicine 2017; 53(5): 719–729. DOI 10.1016/j.amepre.2017.05.011. 28688725

[22] Thornton RLJ , Glover CM , Cené CW , Glik DC , Henderson JA , Williams DR. Evaluating strategies for reducing health disparities by addressing the social determinants of health. Health Affairs 2016; 35(8): 1416–1423. DOI 10.1377/hlthaff.2015.1357. 27503966 PMC5524193

[23] Murray GF , Rodriguez HP , Lewis VA. Upstream with a small paddle: how ACOs are working against the current to meet patients’ social needs. Health Affairs 2020; 39(2): 199–206. DOI 10.1377/hlthaff.2019.01266. 32011930

[24] Purnell TS , Fakunle DO , Bone LR , et al. Overcoming barriers to sustaining health equity interventions: insights from the National Institutes of Health Centers for Population Health and Health Disparities. Journal of Health Care for the Poor and Underserved 2019; 30(3): 1212–1236. DOI 10.1353/hpu.2019.0083. 31422998

[25] Guise NB , Koonce TY , Kusnoor SV , et al. Institute of Medicine measures of social and behavioral determinants of health: a feasibility study. American Journal of Preventive Medicine 2017; 52(2): 199–206. DOI 10.1016/j.amepre.2016.07.033. 27659121 PMC5253326

[26] Adler NE , Stead WW. Patients in context – EHR capture of social and behavioral determinants of health. New England Journal of Medicine 2015; 372(8): 698–701. DOI 10.1056/NEJMp1413945. 25693009

[27] National Association of Community Health Centers (NACHC). Protocol for Responding to and Assessing Patients’ Assets, Risks, and Experiences (PRAPARE) [Internet], 2019 [cited Dec. 13, 2020]. (https://www.nachc.org/research-and-data/prapare/)

[28] Gold R , Cottrell E , Bunce A , et al. Developing electronic health record (EHR) strategies related to health center patients’ social determinants of health. The Journal of the American Board of Family Medicine 2017; 30(4): 428–447. DOI 10.3122/jabfm.2017.04.170046. 28720625 PMC5618800

[29] Barringer-Sterner E. *Building the Foundation for Population Health at OCHIN* [Internet] 2018. [cited Dec. 13, 2020]. (https://ochin.org/blog/population-health-at-ochin)

[30] Gold R , Bunce A , Cottrell E , et al. Study protocol: a pragmatic, stepped-wedge trial of tailored support for implementing social determinants of health documentation/action in community health centers, with realist evaluation. Implementation Science 2019; 14(1): 9. DOI 10.1186/s13012-019-0855-9. 30691480 PMC6348649

[31] Forrest CB , Chesley FD Jr. , Tregear ML , Mistry KB. Development of the learning health system researcher core competencies. Health Services Research 2018; 53(4): 2615–2632. DOI 10.1111/1475-6773.12751. 28777456 PMC6051975

[32] Shenkman EA , Hurt M , Hogan W , et al. OneFlorida Clinical Research Consortium: linking a clinical and translational science institute with a community-based distributive medical education model. Academic Medicine 2018; 93(3): 451–455. DOI 10.1097/ACM.0000000000002029. 29045273 PMC5839715

[33] Harris PA , Taylor R , Minor BL , et al. The REDCap consortium: building and international community of software partners. Journal of Biomedical Informatics 2019; 95: 103208. DOI 10.1016/j.jbi.2019.103208. 31078660 PMC7254481

[34] Sekhon M , Cartwright M , Francis JJ. Acceptability of healthcare interventions: an overview of reviews and development of a theoretical framework. BMC Health Services Research 2017; 17(1): 542. DOI 10.1186/s12913-017-2031-8. 28126032 PMC5267473

[35] Damschroder LJ , Aron DC , Keith RE , Kirsh SR , Alexander JA , Lowery JC. Fostering implementation of health services research findings into practice: a consolidated framework for advancing implementation science. Implementation Science 2009; 4(1): 886. DOI 10.1186/1748-5908-4-50. PMC2736161 19664226

[36] Cady RG , Finkelstein SM. Mixed methods approach for measuring the impact of video telehealth on outpatient clinic triage nurse workflow. Computers, Informatics, Nursing: CIN 2013; 31(9): 439–449. DOI 10.1097/01.NCN.0000432126.99644.6c. PMC4489569 24080753

[37] University of Florida Clinical and Translational Science Institute (CTSI). Citizen Scientist Program [Internet], 2021 [cited Feb. 10, 2021]. (https://www.ctsi.ufl.edu/about/ctsi-programs/about-the-citizen-scientist-program/)

[38] Ayanian JZ , Weissman JS , Schneider EC , et al. Unmet health needs of uninsured adults in the United States. JAMA 2000; 284(16): 2061–2069. DOI 10.1001/jama.284.16.2061. 11042754

[39] Syed ST , Gerber BS , Sharp LK. Traveling towards disease: transportation barriers to health care access. Journal of Community Health 2013; 38(5): 976–993. DOI 10.1007/s10900-013-9681-1. 23543372 PMC4265215

[40] McDermott KW , Jiang HJ. Characteristics and costs of potentially preventable inpatient stays, 2017 [Internet], 2020 [cited Sept, 13, 2021]. 32730017

[41] Warring CD , Pinkney JR , Delvo-Favre ED , et al. Implementation of a routine health literacy assessment at an academic medical center. Journal for Healthcare Quality 2018; 40(5): 247–255. DOI 10.1097/JHQ.0000000000000116. 29166290 PMC6521688

[42] Guest G , Bunce A , Johnson L. How many interviews are enough? An experiment with data saturation and variability. Field Methods 2006; 18(1): 59–82. DOI 10.1177/1525822X05279903.

[43] Hsieh H-F , Shannon SE. Three approaches to qualitative content analysis. Qualitative Health Research 2005; 15(9): 1277–1288. DOI 10.1177/1049732305276687. 16204405

[44] Gale NK , Heath G , Cameron E , Rashid S , Redwood S. Using the framework method for the analysis of qualitative data in multi-disciplinary health research. BMC Medical Research Methodology 2013; 13(1): 117. DOI 10.1186/1471-2288-13-117. 24047204 PMC3848812

[45] Gale RC , Wu J , Erhardt T , et al. Comparison of rapid vs. in-depth analytic methods from a process evaluation of academic detailed in the Veterans Health Administration. Implementation Science 2019; 14(1): 11. DOI 10.1186/s13012-019-0853-y. 30709368 PMC6359833

[46] Averill JB. Matrix analysis as a complementary analytic strategy in qualitative inquiry. Qualitative Health Research 2002; 12(6): 855–866. DOI 10.1177/104973230201200611. 12109729

[47] Berry C , Paul M , Massar R , Marcello RK , Krauskopf M. Social needs screening and referral program at a large US Public Hospital System 2017. American Journal of Public Health 2020; 110(S2): S211–S214. DOI 10.2105/AJPH.2020.305642. 32663088 PMC7362691

[48] Yue D , Pourat N , Chen X , et al. Enabling services improve access to care, preventive services, and satisfaction among health center patients. Health Affairs 2019; 38(9): 1468–1474. DOI 10.1377/hlthaff.2018.05228. 31479374

[49] Easton P , Entwistle VA , Williams B. How the stigma of low literacy can impair patient-professional spoken interactions and affect health: insights from a qualitative investigation. BMC Health Services Research 2013; 13(1): e8364. DOI 10.1186/1472-6963-13-319. PMC3751726 23958036

