# Supplementary material for: Implementation context for addressing social needs in a learning health system: a qualitative study
Source: J Clin Transl Sci. 2021 Aug 31;5(1):e201. doi: 10.1017/cts.2021.842 (PMC8727713; doi:10.1017/cts.2021.842)
Supplement: Supplementary file 1 [file ctssup.zip › S2059866121008426sup001.docx]

**Implementation Strategies to Address Social Determinants of Health and Social Needs (Phase 1)**

Semi-structured patient interview guide

Participant ID: ______ Date: ____________ Start time: _________

Location: ____________________

Interviewer 1: ________________________ Interviewer 2: ________________________

*Thank you for meeting with us today. [Introduce self and Citizen Scientist] We are interested in getting your feedback on a questionnaire that we have developed to help doctors provide better care for their patients. The questionnaire collects information on social and personal factors that have an impact on people’s health, but that doctors do not normally ask about.*

*□ Consent Form Signed*

*□ REALM-SF Completed: Score: _______________*

*I will now turn on the audio recorder. □*

*[Provide iPad.] This tablet has the questionnaire. First, we would like you to complete the questionnaire. You can take as much time as you need. After you have completed the questionnaire, we will ask you about your impressions of the questionnaire and your experience using it.*

PART 1 - Participant completes the Healthy Planet questions on the iPad

- If the participant cannot navigate the module on their own, then make a note of this. In these cases, you may help the user navigate to the screen(s) with the module questions.
- Record the user start time (when they begin using the module) and end time (when they are finished with the module):

□ Completed with assistance □ Completed without assistance

START TIME: ____________ END TIME: ____________

PART 2 – Interview questions

*Now that you have completed the questionnaire, I’d like to ask you about it. There are no right or wrong answers to any of our questions – your opinion is what is most important to us.*

*Anything you say here will be strictly confidential. This interview will take about 20-30 minutes to complete.*

1. What did you think about the questions you were asked? What were your first impressions of these questions?
   1. What ones did you think would be the most helpful to share with your doctor, nurse, social worker or other health professional?
   2. What didn’t you like about the questions?
   3. Does your doctor, nurse, or other health professional often or ever ask you about these kinds of things?
2. Were there any questions that should have been asked and were not? Please describe. [Prompt: remind about study purpose; your social situation, lifestyle or habits]
3. How hard or easy was it for you to answer the questions? [From the perspective of comfort level; if they aren’t sure how to answer, can prompt with “were you comfortable or uncomfortable answering these questions?”]
   1. [If difficult]: What made it difficult for you to answer the questions? [or “What made it uncomfortable to answer or think about?”]
   2. [If easy]: What made it easy for you to answer the questions?
4. How would you feel if you were asked questions like these before you came in for your doctor’s appointments?
5. How often would you like to be asked questions like these? Why?
6. What would be the best way to ask you these questions?

[Prompt: On a tablet or computer? In person? On the phone? Some other way?]

1. Have you heard of the patient portal, called MyChart or MyUFHealth?

[For those that have not heard of it, describe the portal.]

- 1. Would you use the patient portal? Why or why not?
  2. What things might make it difficult for you to use the patient portal?
  3. What about filling out your answers in the waiting room?

1. How do you feel about discussing your answers with your health care provider?
   1. Who would you feel most comfortable discussing your responses with?

[Prompt: A doctor? A nurse? A care manager? Someone else?]

1. Do you think there are any benefits to you or your family to answering the questions? Do you think discussing these topics with your doctor would be helpful for you and your health?
   1. [If yes]: What benefits?

[Prompt: Could it lead to better health? Make it easier to manage chronic conditions?]

- 1. [If no]: Why not?

Thank you so much for your time today! Getting feedback from patients like you will help us design a better experience for all UFHealth patients.

□ Incentive given

_________ End Time
