# Supplementary material for: Implementation context for addressing social needs in a learning health system: a qualitative study
Source: J Clin Transl Sci. 2021 Aug 31;5(1):e201. doi: 10.1017/cts.2021.842 (PMC8727713; doi:10.1017/cts.2021.842)
Supplement: Supplementary file 1 [file ctssup.zip › S2059866121008426sup004.docx]

**Implementation Strategies to Address Social Determinants of Health and Social Needs (Phase 1)**

Provider focus group moderator guide

Number of Participants: Moderator(s):

Date: Note-taker:

Start time: End time:

**WELCOME** (3 min) – [Introduce moderators]

We want to thank you for being here, and appreciate your willingness to share your thoughts with us. Today’s focus group is about the Epic Healthy Planet module that collects information about patients’ social determinants of health, like food, housing, and transportation. The information you give us will be used to improve the way clinics collect and use this information for patients.

Everything we talk about will be considered confidential. This means that we will use the information from this discussion for research but there will not be any names used and no one will know specifically who said what. We also ask that you respect this confidentiality by not sharing other people’s comments outside of the group.

To facilitate this discussion, we will be sharing some screenshots of the module, as well as a workflow diagram for your clinic that was developed in an earlier stage of this study. Although the workflow may not be entirely accurate, it is a good starting place to discuss how to best integrate the module into your clinic. If at any point during the discussion you would like to see these resources again, please do not hesitate to ask us and we’ll put them back up.

**EXPECTATIONS** (2 min)

Now I would like to go over a few agreements to guide our conversation.

- Questions will be posed to the group and it will be your choice if you’d like to answer. At any time, please feel free to pass on a question you don’t feel comfortable answering.
- Please talk **one at a time** and **speak up** as much as possible.
- Because we only have 60 minutes, we may have to shorten the discussion and move on to other questions.
- Feel free to respond to each other about this topic, and not just to me. We’d like this to be more of a discussion than an interview.

**[Using the Zoom application]**

Please take a moment to make sure you have your Zoom display set to gallery view. This will allow you to see everyone in a single view. To enable this, please click “Gallery View” on the top right corner. *[Walk the participants through this if necessary.]*

Please also make sure you are not muted when you want to speak. The mute/unmute button is in the bottom left corner. *[Walk the participant through this if necessary.]*

If at any time you need assistance with using the Zoom application, you can use the Chat function to communicate with us, and we’ll see how we can help.

It’s helpful for us to know everyone’s names during the focus group. I have a list of people who signed up, and would like to do a quick roll call to see who’s here. When I call your name, please let us know if you’re here. [Roll call]

Lastly, in the consent form, we explained that we would like to audio record the conversation and take notes. The notes and the audio will be kept in our office and will not be shared with anyone outside our project. We’ll go ahead and start the audio recording now. *[If any participant objects, remind them that participation is voluntary and they can sign off before we begin.]*

Before we get started, are there any questions?

I. CFIR Intervention Characteristics (10 min)

1. We would like to hear your thoughts about the role that social determinants of health play in contributing to your patients’ health outcomes. [evidence]
2. What do you already know about the Epic Healthy Planet module? [knowledge]
3. Here are some screenshots of what the module looks like for providers and for patients. *[Present screenshots of the EHP module for participants and walk through as needed.]*
4. What do you think about the module? [evidence/attitudes]
5. How easy or difficult do you think it would be to gather information about social determinants of health using the module? [burden/complexity]
6. How easy or difficult do you think it would be to act on the information? [burden/complexity]
7. We interviewed several patients from your clinic about the module, and would like to share some of our findings to get your input.
8. Patients felt that questions dealing with social connections, food, housing, stress, and transportation would be the most helpful to address. Some also suggested adding questions specific to challenges that older patients face, such as fraud and abuse, loneliness, or falling in the home. What are your thoughts about addressing these types of social determinants?

[Probes: Are these difficult questions to ask or discuss? Why/why not?]

[Probes: Is it difficult to give patients assistance with these issues? Why/why not?]

II. CFIR Processes (20 min)

1. Now we’d like to talk about the process for collecting and using information about social determinants in your clinic. This includes things like how often the questions are asked, how the information is collected, and who discusses these issues with patients.
2. For any individual patient, how often do you think these questions should be asked? [Probe: Is there value in administering the module at every visit? Why or why not?]
3. Some patients may be able to complete the module on MyChart at home before arriving for their appointment. However, in our interviews only half of the patients said they were familiar with MyChart, and some expressed concerns about privacy or technical difficulties using it. What has been your experience with promoting the use of MyChart by patients in your clinic? Are there ways we could make this strategy work?
4. Most patients in our interviews had trouble using the module on their own, and they needed assistance completing it. Those who needed help took between 5 and 12 minutes to complete the module. For patients who need help with the module, we’d like your input on how this could be integrated into your clinical workflow. *[Share workflow diagram, walk through as needed.]*
   1. Where would be the most appropriate place in the clinic to administer the module for patients who need help? [Probe: In the waiting room? In the exam room?]
   2. Who would be the most appropriate person to administer the module for patients who need help? [Probe: Nurses? CHWs or social workers?]
5. Patients in our interviews said they preferred to have discussions about social determinants of health with their personal doctors or other trusted providers. Is the clinical encounter with the physician the most appropriate way to discuss these issues with patients? Why or why not?
   1. [If not]: Where, when, and with whom would it be most appropriate to discuss these issues with patients?
6. What should the process be to make referrals based on social determinants of health? [Probes: What resources would you refer patients to? Who would make these referrals? When and how would they make these referrals?]
7. Would you be willing to: (a) participate in a pilot test of these processes?; (b) encourage your patients to do so?

III. CFIR Outer Setting (5 min)

*[NOTE: These questions are only relevant if the participants were already familiar with the Epic SDoH module. Ask if appropriate.]*

1. What incentives are there for you to implement assessment of social determinants using the Epic module? [Prompt: These can be incentives related to making your care of patients more effective, meeting quality metrics, or other things.]
2. What can UF Health and/or FQP do to support you to implement these assessments and referrals for interventions?

IV. CFIR Inner Setting (10 min)

*[NOTE: These questions are worded assuming that clinic leaders / decision-makers are not present. If clinic leaders / decision-makers are present, reword accordingly. E.g., “What do you do to support your staff in efforts to screen for SDoH?”]*

1. How supportive is your clinic setting in efforts to screen for social determinants?
2. [If supportive]: What is the clinic doing to help you in screening?
3. [If not supportive]: What are the barriers?
4. How supportive is your clinic setting in efforts to act upon information about social determinants of health? [Prompt: This could include connecting patients with community resources, providing referrals for care outside the clinic, or other things.]
5. [If supportive]: What is the clinic doing to help you act upon this information?
6. [If not supportive]: What are the barriers?
7. Moving forward, what can your clinic do to help you: (a) assess information on social determinants of health?; (b) act upon information on social determinants of health?

V. CFIR Characteristics of Individuals (5 min)

1. Would you be willing to use the Epic Healthy Planet module with your patients? Why? Why not?
2. Do you think the assessments will make a difference in the outcomes of care for your patients? Why? Why not?

VI. Endpoints (5 min)

1. Moving forward, we are looking at ways to integrate the Epic Healthy Planet module into local clinics for regular use. We will need to measure how effective our efforts are for implementing the module. What things do you think would be most important for us to measure in these efforts?

[Prompt: Some outcomes might be clinician satisfaction, ease of use, patient satisfaction, or others.]

1. What clinical outcomes do you think are most important to evaluate the effectiveness of the intervention to address social determinants of health?

[Prompt: Some clinical outcomes might be patient health care use, clinical endpoints such as Hba1c, or others.]

VII. Closing

That is the end of our questions for today. Would anyone like to share any other comments on the Epic Healthy Planet module or how it can best be implemented in your clinic?
