# Supplementary figures and images for: Implementation context for addressing social needs in a learning health system: a qualitative study
Source: J Clin Transl Sci. 2021 Aug 31;5(1):e201. doi: 10.1017/cts.2021.842 (PMC8727713; doi:10.1017/cts.2021.842)

Clinic 1 workflow

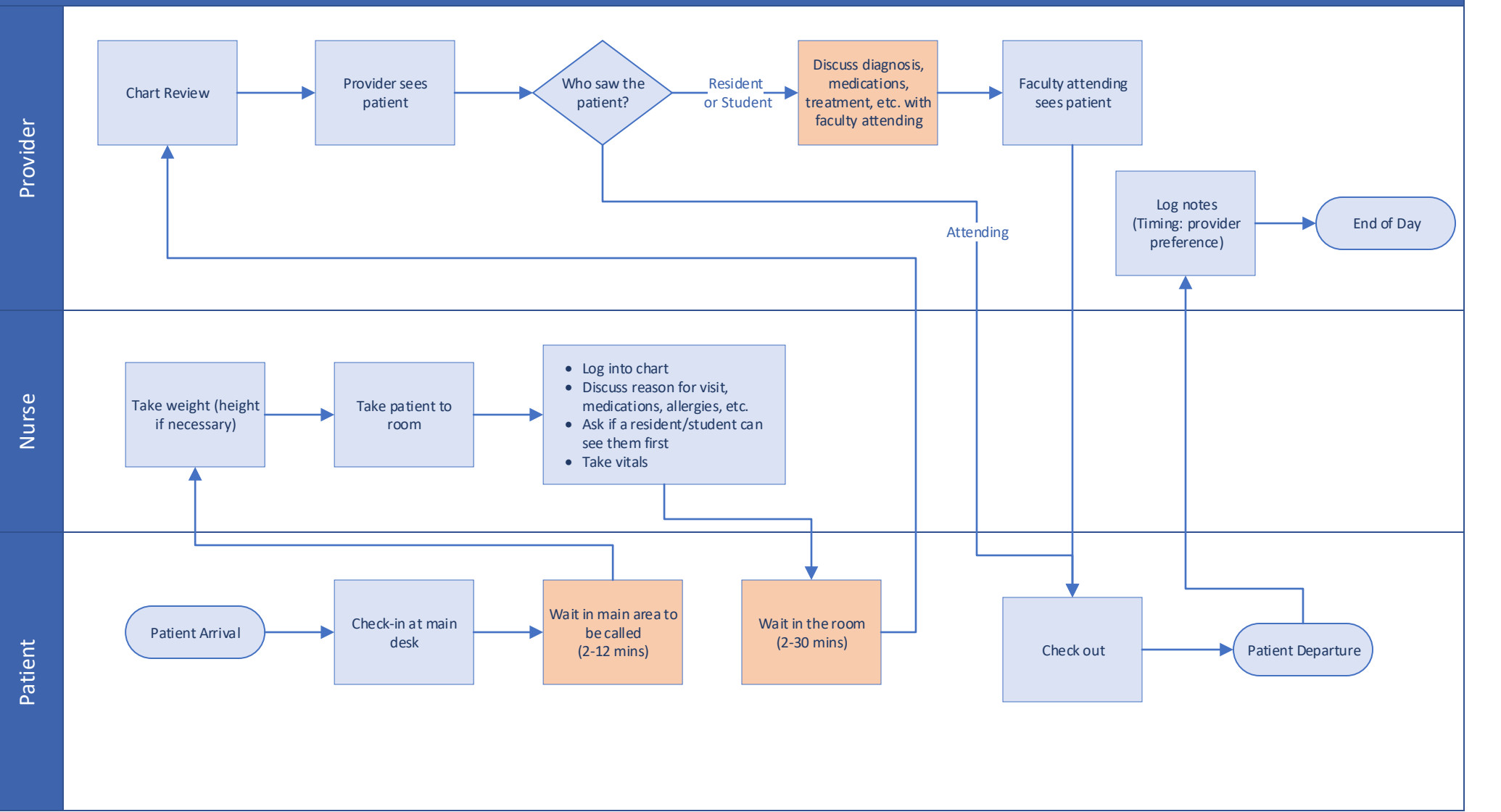

Supplement: Supplementary file 1 [file ctssup.zip › S2059866121008426sup002.pdf]

## Clinic 2 workflow

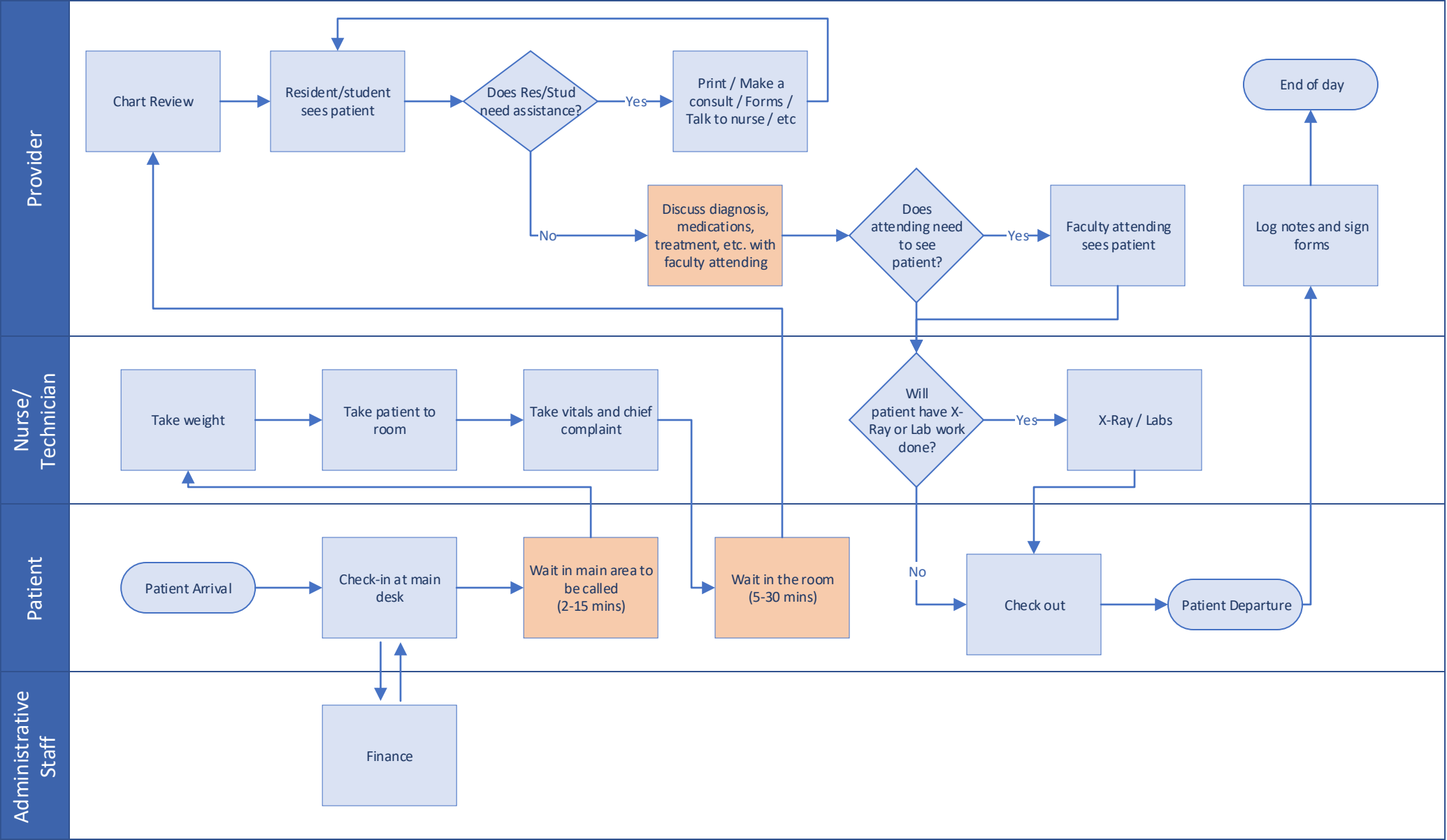

Supplement: Supplementary file 1 [file ctssup.zip › S2059866121008426sup003.pdf]
